# Small Molecule Inhibitors Targeting Nuclear Factor κB Activation Markedly Reduce Expression of Interleukin-2, but Not Interferon-γ, Induced by Phorbol Esters and Calcium Ionophores

**DOI:** 10.3390/ijms222313098

**Published:** 2021-12-03

**Authors:** Yumiko Tanaka, Ayaka Nakao, Yasunobu Miyake, Yukina Higashi, Riho Tanigaki, Takao Kataoka

**Affiliations:** 1 Department of Applied Biology, Kyoto Institute of Technology, Matsugasaki, Sakyo-ku, Kyoto 606-8585, Japan; tymk216@gmail.com (Y.T.); anchoco10@gmail.com (A.N.); yukina77sweet@yahoo.co.jp (Y.H.); tngk.mameuma@gmail.com (R.T.); 2 Department of Biomolecular Sciences, Division of Molecular and Cellular Immunoscience, Faculty of Medicine, Saga University, Saga 849-8501, Japan; ymiyake@cc.saga-u.ac.jp; 3 The Center for Advanced Insect Research Promotion (CAIRP), Kyoto Institute of Technology, Matsugasaki, Sakyo-ku, Kyoto 606-8585, Japan

**Keywords:** IFN-γ, NF-κB, Eomesodermin, NFATc2

## Abstract

The T-box transcription factor Eomesodermin (Eomes) promotes the expression of interferon-γ (IFN-γ). We recently reported that the small molecule inhibitors, TPCA-1 and IKK-16, which target nuclear factor κB (NF-κB) activation, moderately reduced Eomes-dependent IFN-γ expression in mouse lymphoma BW5147 cells stimulated with phorbol 12-myristate 13-acetate (PMA) and ionomycin (IM). In the present study, we investigated the direct effects of NF-κB on IFN-γ expression in mouse lymphoma EL4 cells and primary effector T cells. Eomes strongly promoted IFN-γ expression and the binding of RelA and NFATc2 to the IFN-γ promoter when EL4 cells were stimulated with PMA and IM. Neither TPCA-1 nor IKK-16 reduced IFN-γ expression; however, they markedly decreased interleukin (IL)-2 expression in Eomes-transfected EL4 cells. Moreover, TPCA-1 markedly inhibited the binding of RelA, but not that of Eomes or NFATc2 to the IFN-γ promoter. In effector CD4^+^ and CD8^+^ T cells activated with anti-CD3 and anti-CD28 antibodies, IFN-γ expression induced by PMA and A23187 was not markedly decreased by TPCA-1 or IKK-16 under conditions where IL-2 expression was markedly reduced. Therefore, the present results revealed that NF-κB is dispensable for IFN-γ expression induced by PMA and calcium ionophores in EL4 cells expressing Eomes and primary effector T cells.

## 1. Introduction

Interferon-γ (IFN-γ) is a cytokine that is mainly produced by T helper type 1 (Th1) cells, cytotoxic T cells, natural killer (NK) cells, and other types of cells [1,2]. IFN-γ exerts anti-tumor effects by regulating antigen presentation, the production of cytokines and chemokines, and the promotion of apoptosis pathways [2]. IFN-γ also promotes tumorigenesis via its immunosuppressive effects [3,4]. IFN-γ levels under various physiological and pathogenic conditions are important for maintaining proper immune responses. Naïve CD8^+^ T cells stimulated with an antigen were found to produce low levels of IFN-γ, while high levels of IFN-γ were generated by effector and memory CD8^+^ T cells [5]. The IFN-γ locus in mice is estimated to be approximately 140 kb between CTCF-binding sites [6,7]. In addition to the IFN-γ promoter, the IFN-γ locus possesses conserved non-coding sequences (CNS), which serve as important regulatory sites by transcription factors [8,9,10].

The T-box transcription factors T-bet and Eomesodermin (Eomes) possess a T-box domain, which is necessary for DNA binding, and they are the only T-box family proteins known to regulate the immune system [11]. T-bet has been isolated as a transcription factor that controls the lineage commitment of Th1 cells and their expression of cytokines, such as IFN-γ [12]. Eomes was previously shown to control the function of effector CD8^+^ T cells, including cell-mediated cytotoxicity and the production of perforin, granzyme B, and IFN-γ [13]. The expression of T-bet and Eomes is differently regulated during the differentiation of effector and memory T cells [14]. During CD8^+^ T cell differentiation, T-bet is strongly expressed in effector CD8^+^ T cells, while memory CD8^+^ T cells express low levels of T-bet and high levels of Eomes [15,16,17,18].

T-bet and Eomes are regarded as lineage-specifying transcription factors and epigenetically control the expression of genes such as those encoding cytokines and other transcription factors [8,9,10]. T-bet binds to the IFN-γ promoter and multiple CNS across the IFN-γ locus in T cells and NK cells [7,19,20]. Furthermore, it has been shown to promote permissive histone modifications in IFN-γ promoters and multiple CNS [21,22,23]. Eomes also binds to the IFN-γ promoter in T cells [17,18,24]. In addition to the IFN-γ promoter, we previously demonstrated that Eomes bound to multiple CNS across the IFN-γ locus in mouse lymphoma BW5147 cells [25]. It promoted permissive histone modifications in the IFN-γ promoter and CNS in BW5147 cells [25]. Therefore, Eomes promotes permissive histone modifications to recruit other transcription factors to the IFN-γ promoter and CNS.

A previous study reported that a T cell receptor (TCR) stimulation triggered the activation of nuclear factor of activated T cells (NFAT) and nuclear factor κB (NF-κB) in T cells [26]. The NFAT family is composed of five members, four of which require calcineurin for their transcriptional activation [27]. Gene knockout experiments revealed that calcineurin-dependent NFATc2 (also known as NFAT1) was indispensable for IFN-γ expression in T cells [28,29,30]. NFATc2 interacts with the IFN-γ promoter and CNS [31]. Collectively, these findings reveal that NFATc2 plays an essential role in IFN-γ expression in T cells. The NF-κB family comprises five members (RelA, RelB, c-Rel, p105/p50, and p100/p52), which form heterodimers or homodimers that are capable of DNA binding and transcriptional modulation [32]. As a direct transcriptional activator, RelA was shown to bind to the IFN-γ promoter and multiple IFN-γ CNS [33]. In contrast to this finding, previous studies provided evidence for an indirect role of NF-κB in IFN-γ transcription. The inhibition of NF-κB decreased the expression of T-bet and signal transducer and activator of transcription (STAT) 4 in CD4^+^ T cells [34]. STAT4 was also shown to be involved in the activation of IFN-γ gene expression [8,9,10]. IKK-16, identified as an inhibitor of NF-κB (IκB) kinase [35], reduced the expression of T-bet and Eomes in osteoclast-induced Foxp3^+^ CD25^+^ regulatory CD8^+^ T cells [36]. A TCR stimulation induced Eomes expression in invariant NKT cells [37]. These findings revealed that the activation of NF-κB downstream of a TCR stimulation is required for the expression of multiple transcription factors (e.g., T-bet, Eomes, and STAT4), which are, in turn, essential for the activation of the IFN-γ gene.

We recently reported that RelA interacted with the IFN-γ promoter and multiple CNS in Eomes-transfected BW5147 cells [38]. The dual NF-κB and STAT3 inhibitor, TPCA-1 (2-[(aminocarbonyl)amino]-5-(4-fluorophenyl)-3-thiophenecarboxamide) [39,40], moderately reduced IFN-γ expression induced by phorbol 12-myristate 13-acetate (PMA) and ionomycin (IM), whereas RelA binding to the IFN-γ promoter and CNS was more strongly inhibited by TPCA-1 in Eomes-transfected BW5147 cells [38]. These findings prompted us to verify the necessity of NF-κB for the transcriptional activation of the IFN-γ gene. Therefore, in the present study, we elucidated the role of NF-κB in IFN-γ expression in mouse lymphoma EL4 cells expressing Eomes and effector T cells derived from mouse splenic T cells.

## 2. Results

### 2.1. Eomes Promoted IFN-γ mRNA Expression in EL4 Cells

**Figure 1:**
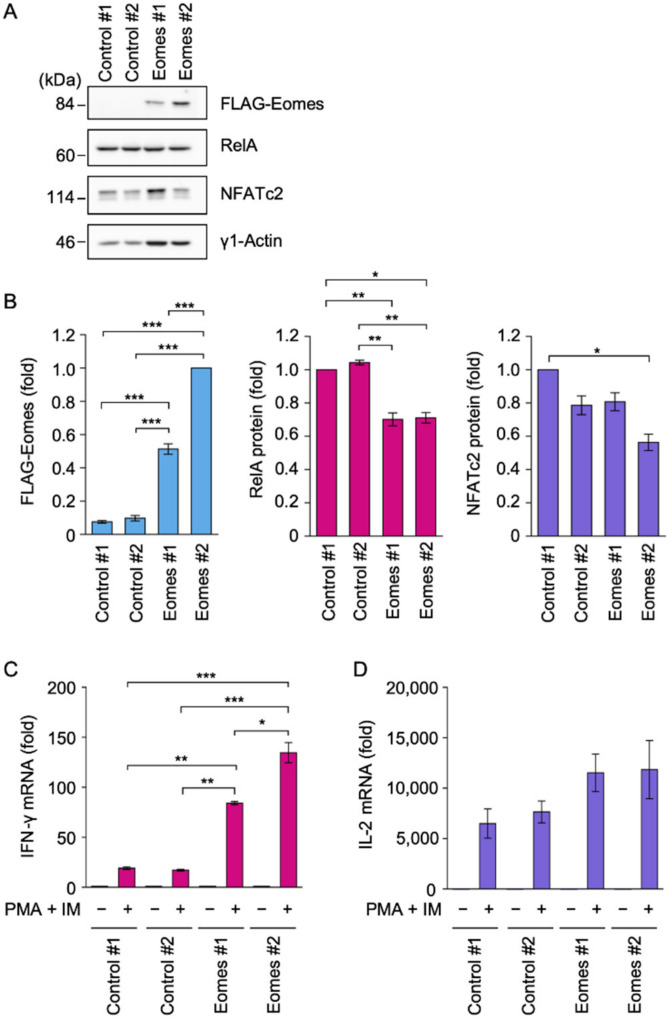
Eomes promoted the expression of IFN-γ mRNA in EL4 cells. (**A**,**B**) Whole cell lysates were prepared from Control #1, Control #2, Eomes #1, and Eomes #2 EL4 transfectants and used for Western blotting using anti-FLAG, anti-RelA, and anti-NFATc2 antibodies. Blots were representative of five independent experiments (**A**). The amount of FLAG-Eomes, RelA, and NFATc2 was normalized to that of γ1-actin. The amount of the FLAG-Eomes protein in the Eomes #2 EL4 transfectant was set to 1-fold. The amounts of the RelA protein and NFATc2 protein in the Control #1 EL4 transfectant were set to 1-fold. The FLAG-Eomes protein (fold), RelA protein (fold), and NFATc2 protein (fold) are shown as the mean ± S.E. of five independent experiments. (**C**,**D**) Control #1, Control #2, Eomes #1, and Eomes #2 EL4 transfectants were treated with (+) or without (−) PMA (10 nM) and IM (1 µM) for 6 h. IFN-γ mRNA (**C**) and IL-2 mRNA (**D**) were measured by quantitative PCR. mRNA levels without PMA and IM in each EL4 transfectant were set to 1-fold. Data are shown as the mean ± S.E. of three independent experiments. * *p* < 0.05, ** *p* < 0.01, and *** *p* < 0.001.

We previously showed that Eomes-transfected EL4 clones produced IFN-γ in response to PMA and IM to mimic the TCR stimulation [25]. In the present study, we established new EL4 clones transfected with control expression vectors or expression vectors encoding elongation factor (EF)-1α promoter-driven FLAG-tagged Eomes. The Eomes #2 transfectant expressed a larger amount of the FLAG-Eomes protein than the Eomes #1 transfectant (Figure 1A,B). Eomes #1 and Eomes #2 transfectants expressed the RelA protein (Figure 1A,B). The NFATc2 protein was also expressed in Eomes #1 and Eomes #2 transfectants (Figure 1A,B).

A schematic of TCR-induced signaling pathways and the experimental design for EL4 transfectants are shown in Figure S1. EL4 transfectants were incubated with PMA and IM for 6 h. The PMA and IM stimulation induced approximately 20-fold increases in IFN-γ mRNA expression in Control #1 and Control #2 transfectants (Figure 1C). In comparison with control transfectants, the PMA and IM stimulation induced approximately 80- and 130-fold increases in IFN-γ mRNA expression in Eomes #1 and Eomes #2 transfectants, respectively (Figure 1C). In contrast, Control #1, Control #2, Eomes #1, and Eomes #2 transfectants expressed interleukin (IL)-2 mRNA at similar levels in response to the PMA and IM stimulation (Figure 1D). Consistent with our previous findings [25], these results confirmed that Eomes strongly promoted IFN-γ mRNA expression in EL4 cells.

### 2.2. Eomes Augmented the Binding of RelA and NFATc2 to the IFN-γ Promoter in EL4 Cells

**Figure 2:**
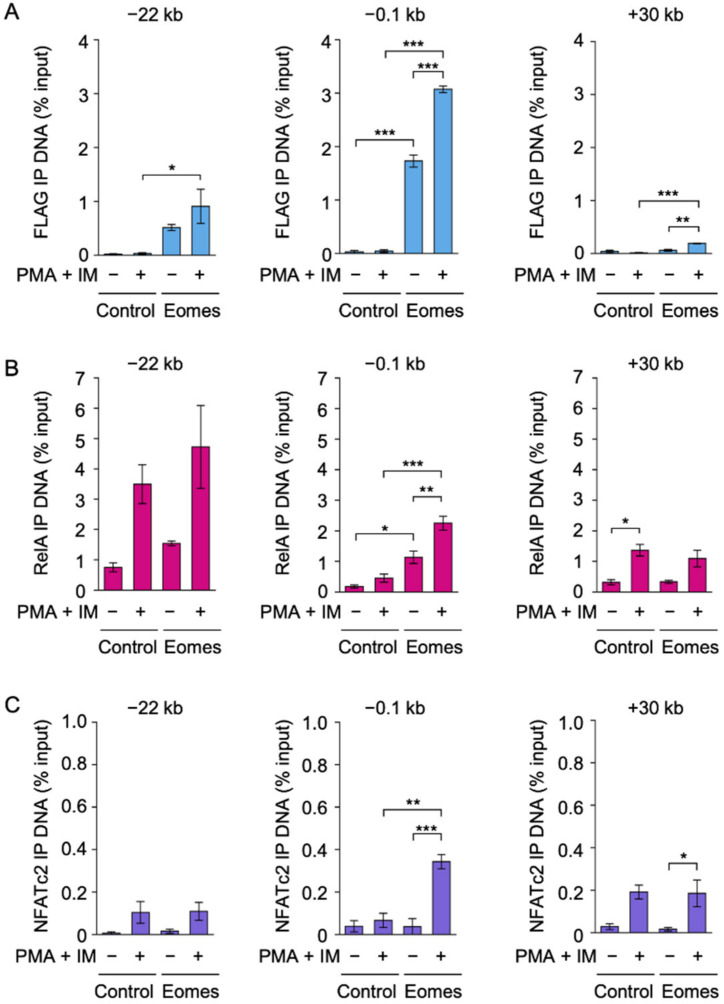
Binding of FLAG-Eomes, RelA, and NFATc2 to the IFN-γ promoter, CNS−22, and CNS+30 in EL4 transfectants. (**A**–**C**) The Control #2 EL4 transfectant (Control) and Eomes #2 EL4 transfectant (Eomes) were treated with (+) or without (−) PMA (10 nM) and IM (1 µM) for 2 h. ChIP assays were performed for FLAG-Eomes (**A**), RelA (**B**), and NFATc2 (**C**). Quantitative PCR was used to measure the amounts of fifteen different DNA regions. The IFN-γ promoter (−0.1 kb), CNS−22, and CNS+30 are shown in this figure. Other regions for FLAG-Eomes, RelA, and NFATc2 are shown in Figures S2–S4, respectively. Immunoprecipitated (IP) DNA (% input) is shown as the mean ± S.E. of three independent experiments. * *p* < 0.05, ** *p* < 0.01, and *** *p* < 0.001.

The IFN-γ promoter and at least nine CNS (−54 kb, −32 kb, −22 kb, −6 kb, +19 kb, +30 kb, +40 kb, +46 kb, and +54 kb) localized between the −71 kb and +67 kb CTCF-binding sites as insulators in the mouse IFN-γ locus [8,9,10]. To investigate the DNA binding of Eomes, Control #2 and Eomes #2 transfectants were incubated with PMA and IM for 2 h, followed by the chromatin immunoprecipitation (ChIP) assay using the anti-FLAG antibody that captures FLAG-Eomes. FLAG-Eomes bound to the IFN-γ promoter at −0.1 kb before and after the PMA and IM stimulation (Figure 2A). Moreover, Eomes interacted with CNS−22 (Figure 2A) and, to a lesser extent, CNS+30 (Figure 2A), but only negligibly with other CNS (Figure S2).

We then investigated the DNA binding of the NF-κB subunit RelA. In the Control #2 transfectant, RelA did not bind to the IFN-γ promoter even when stimulated with PMA and IM (Figure 2B). In contrast, the PMA and IM stimulation markedly increased the binding of RelA at the IFN-γ promoter in the Eomes #2 transfectant (Figure 2B). In the Eomes #2 EL4 transfectant, the PMA and IM stimulation promoted the binding of RelA to CNS−22 and CNS+30 more strongly (*p* values of 0.070 and 0.0561, respectively) than PMA + IM (−) (Figure 2B). Eomes did not markedly affect RelA binding to other CNS in the IFN-γ locus (Figure S3).

NFATc2 binding to the IFN-γ locus was also examined using the ChIP assay. NFATc2 did not bind to the IFN-γ promoter in the Control #2 transfectant (Figure 2C). The PMA and IM stimulation strongly promoted NFATc2 binding to the IFN-γ promoter in the Eomes #2 transfectant (Figure 2C). Eomes did not augment the binding of NFATc2 to IFN-γ CNS (Figure 2C and Figure S4).

### 2.3. TPCA-1 Inhibited PMA- and IM-Induced IL-2 mRNA Expression, but Not IFN-γ mRNA Expression in EL4 Cells

**Figure 3:**
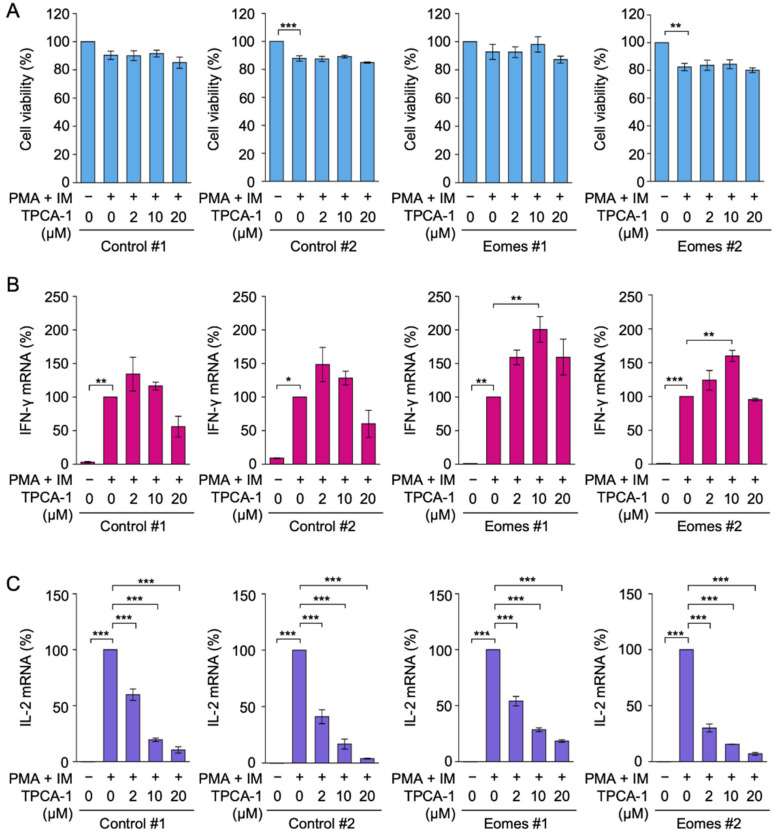
Effects of TPCA-1 on IFN-γ and IL-2 mRNA expression in EL4 transfectants. (**A**–**C**) Control #1, Control #2, Eomes #1, and Eomes #2 EL4 transfectants were pretreated with TPCA-1 for 1 h and then treated with (+) or without (−) PMA (10 nM) and IM (1 µM) for 2 h in the presence or absence of TPCA-1 at the indicated final concentrations. Cell viability (%) is shown as the mean ± S.E. of three independent experiments (**A**). IFN-γ mRNA (**B**) and IL-2 mRNA (**C**) were measured by quantitative PCR. mRNA levels with PMA and IM in each EL4 transfectant were set to 100%. IFN-γ mRNA (%) and IL-2 mRNA (%) are shown as the mean ± S.E. of three independent experiments. * *p* < 0.05, ** *p* < 0.01, and *** *p* < 0.001.

We investigated the involvement of NF-κB in IFN-γ expression in EL4 cells using TPCA-1, which has been used as a dual NF-κB and STAT3 inhibitor [39,40]. To select non-toxic concentrations of TPCA-1, EL4 transfectants were preincubated with or without serial dilutions of TPCA-1 for 1 h and then incubated with PMA and IM for 2 or 6 h. TPCA-1 did not decrease cell viability in the Control or Eomes EL4 transfectants during the 3 h incubation (Figure 3A) or 7 h incubation (Figure S5). It also did not decrease PMA- and IM-induced IFN-γ mRNA expression at concentrations up to 20 µM in Eomes #1 and Eomes #2 transfectants (Figure 3B). TPCA-1 at 10 µM increased the expression of IFN-γ mRNA in Eomes #1 and Eomes #2 transfectants (Figure 3B). In contrast, TPCA-1 decreased PMA- and IM-induced IL-2 mRNA expression in a dose-dependent manner and at concentrations higher than 2 µM in Control #1, Control #2, Eomes #1, and Eomes #2 transfectants (Figure 3C).

### 2.4. IKK-16 Inhibited PMA- and IM-Induced IL-2 mRNA Expression, but Not IFN-γ mRNA Expression in EL4 Cells

**Figure 4:**
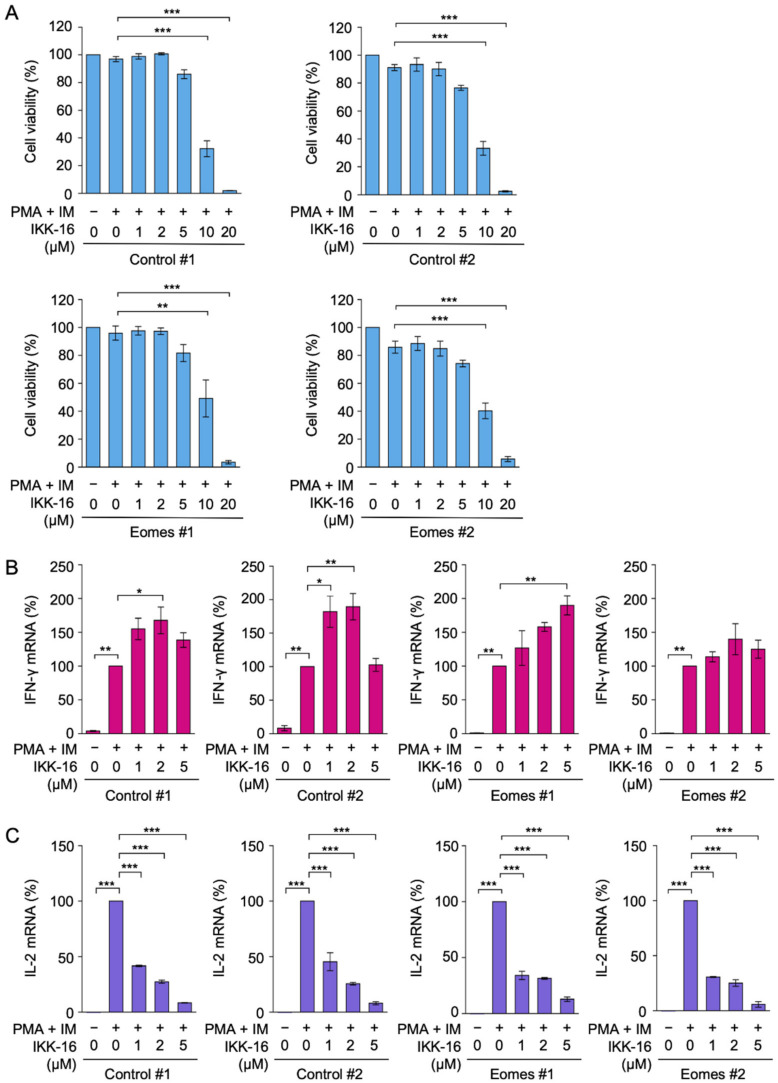
Effects of IKK-16 on IFN-γ and IL-2 mRNA expression in EL4 transfectants. (**A**–**C**) Control #1, Control #2, Eomes #1, and Eomes #2 EL4 transfectants were pretreated with IKK-16 for 1 h and then treated with (+) or without (−) PMA (10 nM) and IM (1 µM) for 2 h in the presence or absence of IKK-16 at the indicated final concentrations. Cell viability (%) is shown as the mean ± S.E. of three independent experiments (**A**). IFN-γ mRNA (**B**) and IL-2 mRNA (**C**) were measured by quantitative PCR. mRNA levels with PMA and IM in each EL4 transfectant were set to 100%. IFN-γ mRNA (%) and IL-2 mRNA (%) are shown as the mean ± S.E. of three independent experiments. * *p* < 0.05, ** *p* < 0.01, and *** *p* < 0.001.

To confirm the above results, we additionally used IKK-16, which has been identified and employed as a selective IκB kinase inhibitor [35]. IKK-16 markedly decreased cell viability at concentrations higher than 10 µM during the 3 h incubation in all EL4 transfectants (Figure 4A), while IKK-16 at concentrations up to 5 µM did not decrease cell viability even during the 7 h incubation (Figure S6).

IKK-16 did not decrease PMA- and IM-induced IFN-γ mRNA expression at concentrations up to 5 µM in Eomes #1 and Eomes #2 transfectants (Figure 4B). It appeared to augment the expression of IFN-γ mRNA at particular concentrations in Control #1, Control #2, and Eomes #1 transfectants (Figure 4B). In contrast, IKK-16 markedly inhibited IL-2 mRNA expression at concentrations higher than 1 µM (Figure 4C). Collectively, these results indicate that TPCA-1 and IKK-16 did not reduce IFN-γ mRNA expression in EL4 cells even under concentrations that markedly inhibited IL-2 mRNA expression.

### 2.5. TPCA-1 Inhibited the Binding of RelA, but Not NFATc2 or Eomes, to the IFN-γ Promoter in EL4 Cells

**Figure 5:**
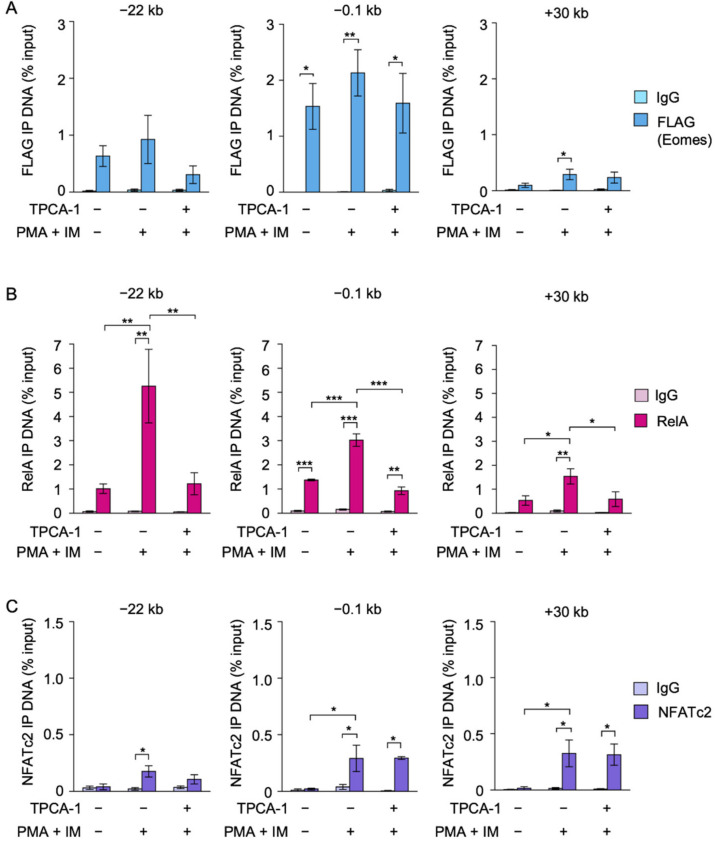
Effects of TPCA-1 on the binding of RelA, FLAG-Eomes, and NFATc2 to the IFN-γ promoter, CNS−22, and CNS+30 in EL4 transfectants. (**A**–**C**) Eomes #2 EL4 transfectants were pretreated with (+) or without (−) TPCA-1 for 1 h and then treated with (+) or without (−) PMA (10 nM) and IM (1 µM) for 2 h in the presence or absence of TPCA-1 (20 µM). ChIP assays were performed for FLAG-Eomes (**A**), RelA (**B**), and NFATc2 (**C**) using control IgG (light color bars) or specific antibodies (dark color bars). Quantitative PCR was used to measure the amounts of DNA regions. Immunoprecipitated (IP) DNA (% input) is shown as the mean ± S.E. of four (**A**) and three (**B**,**C**) independent experiments. * *p* < 0.05, ** *p* < 0.01, and *** *p* < 0.001.

The DNA binding of FLAG-Eomes, RelA, and NFATc2 in Eomes #2 EL4 transfectants was investigated by the ChIP assay. FLAG-Eomes bound to the IFN-γ promoter before and after the PMA and IM stimulation (Figure 5A). The PMA and IM stimulation did not appear to promote the binding of FLAG-Eomes to the IFN-γ promoter (Figure 5A). TPCA-1 did not decrease the binding of FLAG-Eomes to the IFN-γ promoter (Figure 5A).

In response to the PMA and IM stimulation, RelA binding to the IFN-γ promoter, CNS−22, and CNS+30 markedly increased in the Eomes #2 transfectant (Figure 5B). TPCA-1 reduced the amount of RelA binding to the IFN-γ promoter, CNS−22, and CNS+30 to unstimulated levels (Figure 5B).

The PMA and IM stimulation augmented the binding of NFATc2 to the IFN-γ promoter and CNS+30 (Figure 5C). However, TPCA-1 did not markedly reduce the binding of NFATc2 to the IFN-γ promoter and CNS+30 (Figure 5C). Taken together, these results showed that TPCA-1 effectively prevented the binding to RelA to the IFN-γ promoter, but did not diminish the binding of FLAG-Eomes and NFATc2 to the IFN-γ promoter in EL4 cells.

### 2.6. TPCA-1 and IKK-16 Inhibited IL-2 Expression, but Not IFN-γ Expression in Primary Effector CD4^+^ and CD8^+^ T Cells

**Figure 6:**
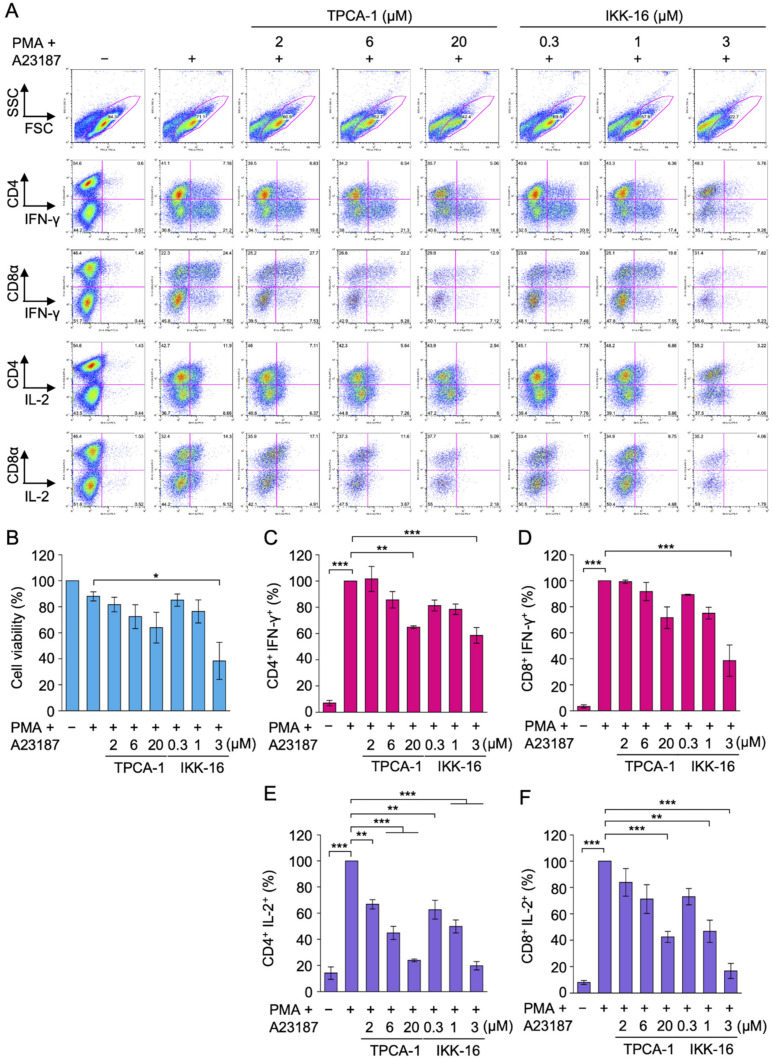
Effects of TPCA-1 and IKK-16 on IFN-γ and IL-2 expression in primary effector T cells. (**A**–**F**) Splenic T cells were purified and incubated with anti-CD3 and anti-CD28 antibodies for 3 d. T cells were preincubated with the indicated concentrations of TPCA-1 or IKK-16 for 1 h and then incubated with (+) or without (−) PMA (20 ng/mL), A23187 (0.5 µg/mL), and monensin (2 µM) for 4 h in the presence or absence of TPCA-1 or IKK-16. Cells were stained with anti-CD4-allophycocyanin (APC) or anti-CD8α-APC, fixed and permeabilized, followed by staining with anti-IFN-γ-fluorescein isothiocyanate (FITC) or anti-IL-2-phycoerythrin (PE). (**A**) Plots are representative of three independent experiments performed in triplicate. Viable cells were surrounded by a red line in FSC-SSC plots. (**B**) Cell viability without PMA and A23187 was set to 100%. Cell viability (%) is shown as the mean ± S.E. of three independent experiments. (**C** to **F**) IFN-γ and IL-2 expression with PMA and A23187 was set to 100%. The percentage of CD4^+^ IFN-γ^+^ cells (**C**), CD8^+^ IFN-γ^+^ cells (**D**), CD4^+^ IL-2^+^ cells (**E**), and CD8^+^ IL-2^+^ cells (**F**) is shown as the mean ± S.E. of three independent experiments. * *p* < 0.05, ** *p* < 0.01, and *** *p* < 0.001.

The present results demonstrated that TPCA-1 and IKK-16 did not inhibit IFN-γ expression at concentrations that markedly suppressed IL-2 expression in Eomes-transfected EL4 cells stimulated with PMA and IM. We also previously showed that TPCA-1 and IKK-16 moderately reduced IFN-γ expression in Eomes-transfected BW5147 cells [38]. To generalize these findings, primary T cells were prepared from mouse spleens and used in experiments. An experimental design for primary effector T cells is shown in Figure S7. Naïve T cells were activated by anti-CD3 and anti-CD28 antibodies for 3 d. Effector CD4^+^ and CD8^+^ T cells were capable of producing detectable amounts of IFN-γ and IL-2 following a 4 h exposure to PMA and A23187 (calcium ionophore) (Figure 6A). Viable cells and non-viable cells were distinguished by two-dimensional patterns of forward and side scatters (Figure 6A).

Effector CD4^+^ and CD8^+^ T cells were preincubated with serial dilutions of TPCA-1 and IKK-16 for 1 h and then incubated with PMA and A23187 for 4 h. TPCA-1 slightly reduced cell viability as its concentration increased (Figure 6B). TPCA-1 also slightly decreased IFN-γ expression in CD4^+^ and CD8^+^ T cells (Figure 6C,D). IKK-16 at 3 µM reduced cell viability by approximately 60% (Figure 6B). In CD4^+^ and CD8^+^ T cells, IKK-16 at 3 µM decreased IFN-γ expression by approximately 40 and 60%, respectively (Figure 6C,D). Therefore, IKK-16-brought decreases in IFN-γ expression appeared to be mainly attributed to reductions in cell viability. In contrast to IFN-γ expression, TPCA-1 markedly reduced IL-2 expression at 2–20 µM in CD4^+^ T cells and at 20 µM in CD8^+^ T cells (Figure 6E,F). IKK-16 at 0.3 or 1 µM decreased IL-2 expression in CD4^+^ T cells and CD8^+^ T cells (Figure 6E,F). These results indicate that TPCA-1 and IKK-16 did not inhibit IFN-γ expression even under concentrations that markedly inhibited IL-2 expression in primary effector CD4^+^ and CD8^+^ T cells.

## 3. Discussion

Eomes is a transcription factor that specifies the lineage of T cells and NK cells [11,14,41]. The ectopic expression of Eomes confers IFN-γ expression to several types of normal and transformed cells [13,24,42,43,44]. Consistent with these findings, we also reported that the transfection of Eomes promoted IFN-γ expression in mouse lymphoma BW5147 and EL4 cells, neither of which expressed detectable amounts of endogenous Eomes [25,38]. Stimulation with TCR induced Eomes expression in invariant NKT cells [37]. Furthermore, IL-2 and IL-12 promoted the transcription of Eomes [45]. Collectively, these findings indicated that Eomes is transcriptionally regulated by TCR and induces to produce cytokines. To avoid an indirect influence of Eomes levels, the BW5147 and EL4 transfectants used in our studies provide ideal model systems for Eomes-dependent transcription because Eomes is constitutively expressed under the control of the housekeeping EF-1α promoter.

In addition to T-box sites [46], the IFN-γ promoter contains binding sites for NF-κB and NFAT [47]. We previously showed that IFN-γ expression induced by PMA and IM in Eomes-transfected BW5147 cells and EL4 cells was markedly inhibited by FK506 [25,38]. This finding indicated that the calcineurin-NFAT pathway is indispensable for Eomes-dependent IFN-γ expression. In contrast, TPCA-1 and IKK-16 moderately decreased IFN-γ expression induced by PMA and IM in Eomes-transfected BW5147 cells, whereas IL-2 expression was more strongly inhibited [38]. In the present study, TPCA-1 and IKK-16 did not inhibit PMA- and IM-induced IFN-γ expression in Eomes-transfected EL4 cells, while TPCA-1 effectively blocked the binding of RelA to the IFN-γ promoter, CNS−22, and CNS+30 in Eomes-transfected EL4 cells. A previous study reported that IKK-16 reduced the expression of Eomes or T-bet, and NF-κB was found to be enriched in the Eomes and T-bet promoters in osteoclast-induced Foxp3^+^ CD25^+^ regulatory CD8^+^ T cells [36]. These findings indicate that NF-κB plays an auxiliary or dispensable role in PMA- and IM-induced IFN-γ transcription when Eomes or T-bet is expressed. Based on the present results, we propose that in the first phase, NF-κB is required for the expression of Eomes or T-bet, and in the second phase, they epigenetically activate the IFN-γ gene to allow the access of other transcription factors, such as NFATc2. In contrast to Eomes-transfected BW5147 cells, TPCA-1 appeared to increase IFN-γ expression in Eomes-transfected EL4 cells. We speculate that this was due to NF-κB subunits expressed in EL4 cells, but not BW5147 cells, negatively regulating the transcription of the IFN-γ gene.

We mainly investigated two mouse lymphoma cell lines stably expressing Eomes to elucidate the mechanisms underlying IFN-γ transcription. To generalize our hypothesis, we also examined primary T cells. In response to stimulation with TCR, naïve T cells produce IFN-γ at low levels, whereas effector T cells and memory T cells produce higher levels of IFN-γ [5]. In CD8^+^ T cell differentiation, the expression of T-bet and Eomes is induced in the early and late phases, respectively, upon stimulation with anti-CD3 and anti-CD28 antibodies [17,18]. High IL-2 levels have been shown to up-regulate the expression of Eomes in CD8^+^ T cells [18]. IL-2 and IL-12 have been reported to promote the transcription of Eomes [45]. Therefore, during the differentiation of naïve T cells into effector T cells, T-bet, or possibly Eomes, epigenetically activates the IFN-γ gene by histone modifications. Effector T cells harbor T-bet, or possibly Eomes, at sufficient levels and are primed to produce IFN-γ immediately upon stimulation with TCR. Since neither TPCA-1 nor IKK-16 markedly inhibited IFN-γ expression induced by PMA and A23187 in effector CD4^+^ and CD8^+^ T cells, we concluded that NF-κB is dispensable for IFN-γ transcription in effector T cells stimulated with phorbol esters and calcium ionophores.

IL-18 belongs to the IL-1 family and has been reported to induce IFN-γ expression in T cells and NK cells [48,49]. Upon binding to IL-18, IL-18 receptors form αβ heterodimers, which mediate the MyD88-dependent activation of IκB kinase, leading to the activation of NF-κB [48,49]. Stimulations with IL-12 and IL-18 were shown to induce IFN-γ expression, which was accompanied by the recruitment of RelA to multiple CNS in the IFN-γ locus in Th1 and effector CD8^+^ T cells [33]. IL-18, particularly in combination with IL-12, was also found to augment IFN-γ production in T cells and NK cells [49]. Toll-like receptor 2 (TLR2) is known to induce the MyD88-dependent activation of NF-κB [50]. Stimulation with TLR2 induced IFN-γ production in effector Th1 cells [51]. In contrast to the Toll-IL-1 receptor family, TCR-dependent NF-κB activation requires the distinct signaling proteins CARMA1, BCL10, and MALT1 [52]. Therefore, NF-κB may be a more dominant activator of IFN-γ transcription when stimulated with IL-18 or TLR2 ligands.

The ChIP assay showed that Eomes bound to the IFN-γ promoter, CNS−22, and CNS+30 in Eomes-transfected EL4 cells. Among regulatory regions across the IFN-γ locus, the IFN-γ promoter appears to be critical for Eomes-dependent activation because the binding of NFATc2 and RelA to the IFN-γ promoter was markedly augmented only when EL4 cells were transfected with Eomes and then stimulated with PMA and IM. TPCA-1 did not markedly prevent the binding of Eomes or NFATc2 to the IFN-γ promoter or Eomes-dependent IFN-γ transcription in EL4 cells. We previously showed that FK506 inhibited PMA- and IM-induced IFN-γ expression and the binding of Eomes to the IFN-γ promoter in Eomes-transfected EL4 cells [25]. Therefore, in contrast to RelA, the binding of NFATc2 to the IFN-γ promoter appears to be indispensable for IFN-γ expression in EL4 cells. 

## 4. Materials and Methods

### 4.1. Mice

Female C57BL/6N mice between 6 and 8 weeks old were purchased from Kyudo Co. (Tosu, Japan). All animal experiments were approved by the Animal Care and Use Committee for Saga University.

### 4.2. Cells

Mouse lymphoma EL4 cells (RCB1641) were obtained from the RIKEN BioResource Research Center (Tsukuba, Japan). EL4 cells were maintained in RPMI 1640 medium supplemented with heat-inactivated fetal calf serum (Sigma-Aldrich, St. Louis, MO, USA) and a penicillin–streptomycin mixture (Nacalai Tesque, Kyoto, Japan). 

### 4.3. Reagents

PMA (FUJIFILM Wako Pure Chemical Corporation, Osaka, Japan), IM (Merck Millipore, Darmstadt, Germany), TPCA-1 (Sigma-Aldrich, St. Louis, MO, USA), IKK-16 dihydrochloride (Sigma-Aldrich, St. Louis, MO, USA), A23187 (Sigma-Aldrich, St. Louis, MO, USA), and monensin (Sigma-Aldrich, St. Louis, MO, USA) were used for experiments.

### 4.4. Antibodies

Primary antibodies to γ1-actin (013-24553, Clone No. 2F3; FUJIFILM Wako Pure Chemical Corporation, Osaka, Japan), FLAG (012-22384, Clone No. 1E6; FUJIFILM Wako Pure Chemical Corporation, Osaka, Japan), NFATc2 (sc-7296, Clone No. 4G6-G5; Santa Cruz Biotechnology, Dallas, TX, USA), and RelA (sc-372, C-20; Santa Cruz Biotechnology, Dallas, TX, USA) were used for Western blotting and ChIP assays. A peroxidase-conjugated anti-mouse IgG antibody (115-035-146; Jackson ImmunoResearch, West Grove, PA, USA) and peroxidase-conjugated anti-rabbit IgG antibody (111-035-144; Jackson ImmunoResearch, West Grove, PA, USA) were used for Western blotting. Mouse IgG2a (400201, MOPC-173; BioLegend, San Diego, CA, USA), mouse IgG2b (400301, MPC-11; BioLegend, San Diego, CA, USA), and rabbit IgG (PP64; Merck Millipore, Darmstadt, Germany) were used as control antibodies in the ChIP assay. An APC-conjugated anti-CD4 antibody (17-0041-82, GK1.5; eBioscience, San Diego, CA, USA), APC-conjugated anti-CD8α antibody (MA1-10302, 53-6.7; eBioscience, San Diego, CA, USA), FITC-conjugated anti-IFN-γ antibody (11-7311-82, XMG1.2; eBioscience, San Diego, CA, USA), and PE-conjugated anti-IL-2 antibody (503807, JES6-5H4; BioLegend, San Diego, CA, USA) were used for flow cytometry.

### 4.5. Transfection

EL4 cells were transfected with an EF-1α promoter-driven pEF pGKpuro expression vector (a kind gift from Dr. D.C.S. Huang) [53] or pEF pGKpuro expression vector encoding N-terminal FLAG-tagged mouse Eomes [25] using the GenePulser Xcell electroporation system (Bio-Rad Laboratories, Hercules, CA, USA). Transfected cells were cultured in the presence of puromycin (5 µM). Puromycin-resistant clones were isolated independently and further cultured in the presence of puromycin. Control #1, Control #2, Eomes #1, and Eomes #2 EL4 transfectants were selected and used in the present study.

### 4.6. Western Blotting

Whole cell lysates were prepared and analyzed by Western blotting as previously described [54]. The anti-γ1-actin antibody (0.5 mg/mL, 1/1000 dilution), anti-FLAG antibody (0.5 mg/mL, 1/1000 dilution), anti-NFATc2 antibody (200 µg/mL, 1/1000 dilution), anti-RelA antibody (100 µg/mL, 1/1000 dilution), peroxidase-conjugated anti-mouse IgG antibody (0.8 mg/mL, 1/5000 dilution), and peroxidase-conjugated anti-rabbit IgG antibody (0.8 mg/mL, 1/5000 dilution) were used. γ1-Actin was used as a loading control for human and mouse cell lines in our previous studies [38,54]. Blots were visualized and acquired by Amersham Imager 680 (GE Healthcare Japan, Tokyo, Japan). ImageQuant TL software (GE Healthcare Japan, Tokyo, Japan) was used to quantitate protein bands. 

### 4.7. Quantitative PCR

Total RNA preparation and cDNA synthesis were performed as previously described [55]. mRNA levels were measured by Thermal Cycler Dice^®^ Real Time System Lite (Takara Bio, Kusatsu, Japan) using primers for IFN-γ [56], IL-2 [56], and β-actin [57]. Primer-specific standard curves were used for the quantitation of initial DNA.

### 4.8. ChIP Assay

The ChIP assay was performed as previously described [25]. Immunoprecipitation was conducted with 1 µg of the anti-FLAG antibody (1E6; mouse IgG2b), control mouse IgG2b (MPC-11), anti-NFATc2 antibody (4G6-G5; mouse IgG2a), control mouse IgG2a (MOPC-173), anti-RelA antibody (C-20; rabbit IgG), or control rabbit IgG (PP64). Primers used for the quantitation of DNA regions in the IFN-γ locus were previously described [25].

### 4.9. Cell Viability Assay

Cell viability was evaluated based on the formazan formation of 3-(4,5-dimethyl-2-thiazolyl)-2,5-diphenyltetrazolium bromide as previously described [58,59], except for the use of the iMark microplate reader (Bio-Rad Laboratories, Hercules, CA, USA).

### 4.10. Purification and Culture of Primary T Cells

The spleen was minced with scissors, treated with RBC lysis buffer (420301, BioLegend, San Diego, CA, USA) for 2 min, and filtered through a 70 µm nylon mesh. Spleen cells were then treated with FITC-conjugated antibodies reactive to B220 (103205, RA3-6B2; BioLegend, San Diego, CA, USA), CD11b (101205, M1/70; BioLegend, San Diego, CA, USA), and CD11c (117305, N418; BioLegend, San Diego, CA, USA), followed by treatment with anti-FITC antibody-conjugated magnetic beads (130-048-701, Miltenyi Biotec, Bergisch Gladbach, Germany). Primary T cells were purified by negative sorting using a MACS LD column (Miltenyi Biotec, Bergisch Gladbach, Germany), and incubated in plates coated with 10 µg/mL of the anti-CD3 antibody (100301, 145-2C11; BioLegend, San Diego, CA, USA) and 2 µg/mL of the anti-CD28 antibody (16-0281-82, 37.51; eBioscience, San Diego, CA, USA) for 3 d. The purity of T cells was routinely up to 90%, as assessed by anti-CD3ε staining.

### 4.11. Intracellular Staining

T cells were incubated with monensin (2 µM) during the PMA and A23187 stimulation to prevent cytokine secretion. Cells were harvested and stained with APC-conjugated antibodies for CD4 and anti-CD8α, and treated with Fixation/Permeabilization solution (BD Biosciences, San Jose, CA, USA). Cells were further stained with the FITC-conjugated anti-IFN-γ antibody or PE-conjugated anti-IL-2 antibody. Stained cells were analyzed by FACSCalibur (BD Biosciences, San Jose, CA, USA). Plots were analyzed by FlowJo software (Tomy Digital Biology, Tokyo, Japan).

### 4.12. Statistical Analysis

Statistical analyses were performed using a one-way ANOVA and Tukey’s test. 

## 5. Conclusions

We herein revealed that NF-κB is dispensable for IFN-γ expression induced by phorbol esters and calcium ionophores in EL4 cells expressing Eomes. This is also applicable to primary effector CD4^+^ and CD8^+^ T cells, which are capable of rapidly producing IFN-γ in response to stimulation with TCR. In addition to the TCR stimulation, the NF-κB signaling pathway is induced by inflammatory cytokines and TLR ligands. Therefore, the requirement of NF-κB as a direct activator of IFN-γ transcription in response to a TCR stimulation appears to depend on the intracellular levels of pre-existing transcription factors required for epigenetic modulations in the IFN-γ gene and the strength of NF-κB signals by extracellular ligands.

## Data Availability

The data presented in the present study are available upon request from the corresponding author.

## Supplementary Materials

The following are available online at https://www.mdpi.com/article/10.3390/ijms222313098/s1.
