# Supplementary material for: Small Molecule Inhibitors Targeting Nuclear Factor κB Activation Markedly Reduce Expression of Interleukin-2, but Not Interferon-γ, Induced by Phorbol Esters and Calcium Ionophores
Source: Int J Mol Sci. 2021 Dec 3;22(23):13098. doi: 10.3390/ijms222313098 (PMC8658103; doi:10.3390/ijms222313098)
Supplement: Supplementary file 1 [file ijms-22-13098-s001.zip › ijms-1470211-supplementary.pdf]

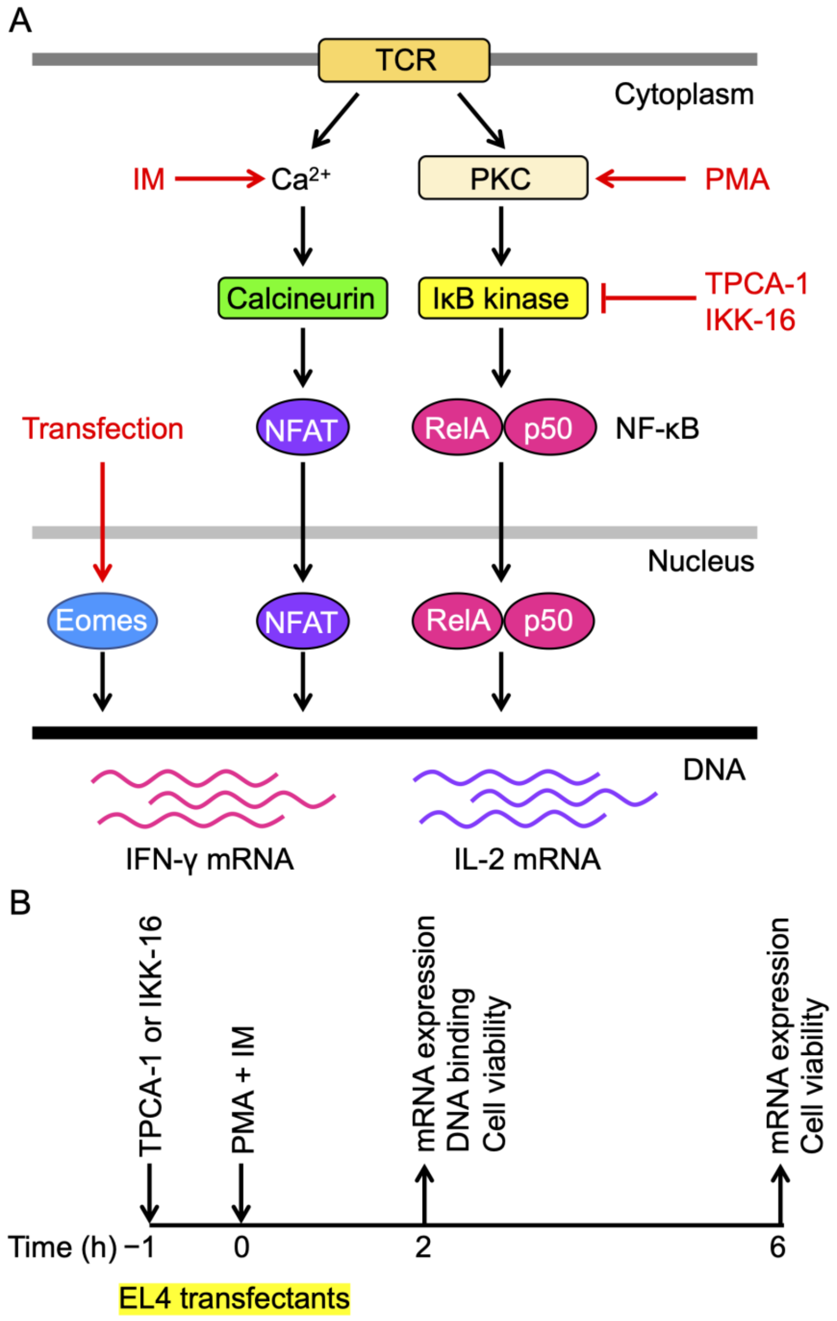

Figure S1. A schematic of TCR-induced signaling pathways and the experimental design for EL4 transfectants. (A) The TCR stimulation induces the activation of protein kinase C (PKC) and augments the level of intracellular Ca^{2+}, which ultimately promotes the transcription of cytokines, such as IFN-γ and IL-2. NF-κB and NFAT are transcription factors that are activated upon a TCR stimulation. PMA and IM are used to mimic the TCR stimulation. PMA activates PKC, and ultimately induces the activation of IκB kinase, which phosphorylates IκB, leading

to its proteasomal degradation and the liberation of NF-κB heterodimers, such as those consisting of RelA and p50. IM is a calcium ionophore that increases the level of intracellular Ca^{2+}, and thereby activates calcineurin, which dephosphorylates NFAT isoforms, allowing their translocation to the nucleus. TPCA-1 and IKK-16 inhibit IκB kinase in the NF-κB signaling pathway. Mouse lymphoma EL4 cells were stably transfected with an expression vector encoding Eomes or a control expression vector. (B) EL4 transfectants were pretreated with TPCA-1 or IKK-16 for 1 h, and then treated with or without PMA (10 nM) plus IM (1 μM) for the indicated times in the presence or absence of TPCA-1 or IKK-16 to measure mRNA expression, DNA binding, or cell viability.

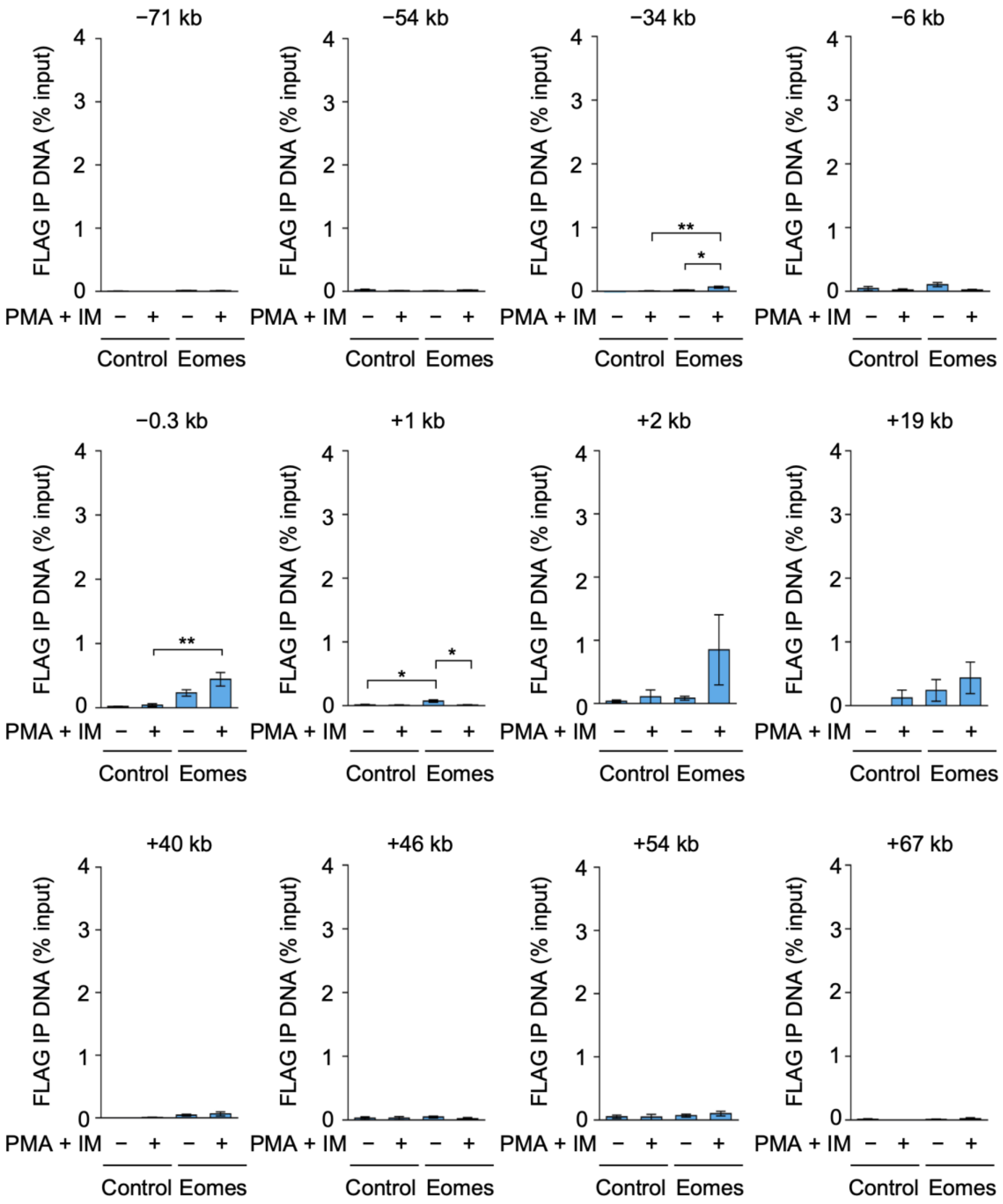

Figure S2. Binding of FLAG-Eomes to specific regions across the IFN-γ locus in EL4 transfectants. Regarding experimental details and other regions, refer to Figure 2A.

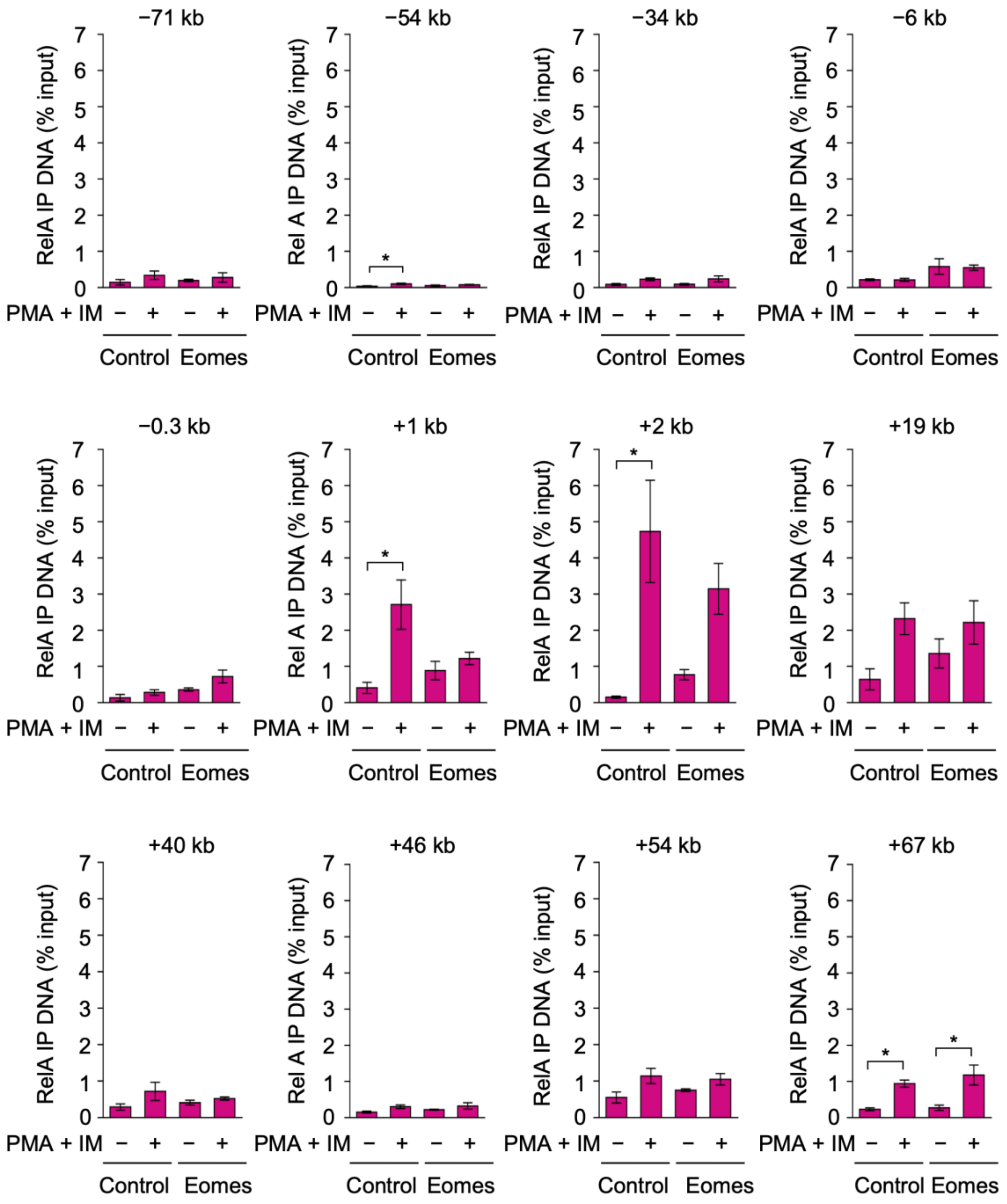

Figure S3. Binding of RelA to specific regions across the IFN-γ locus in EL4 transfectants. Regarding experimental details and other regions, refer to Figure 2B.

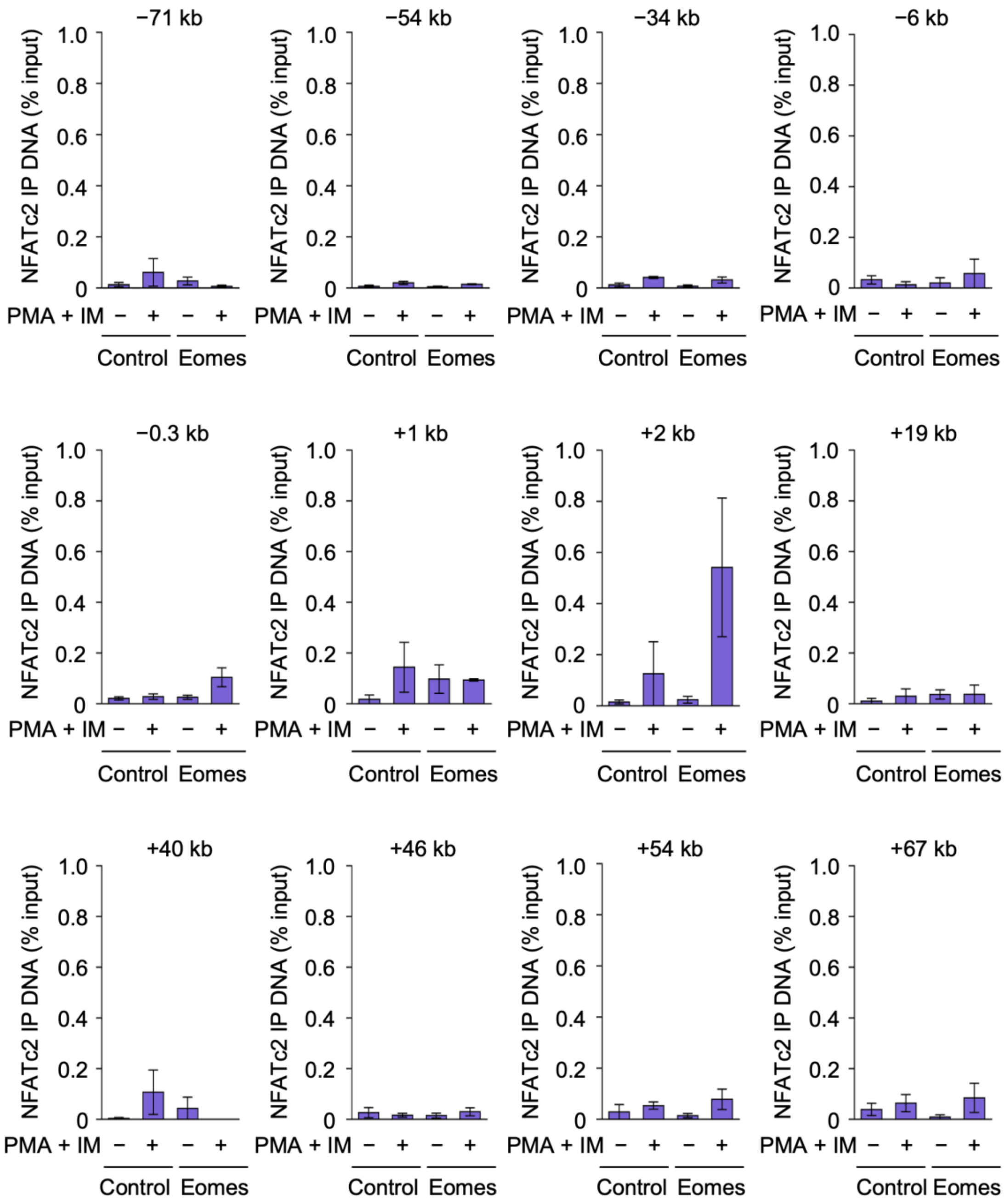

Figure S4. Binding of NFATc2 to specific regions across the IFN-γ locus in EL4 transfectants. Regarding experimental details and other regions, refer to Figure 2C.

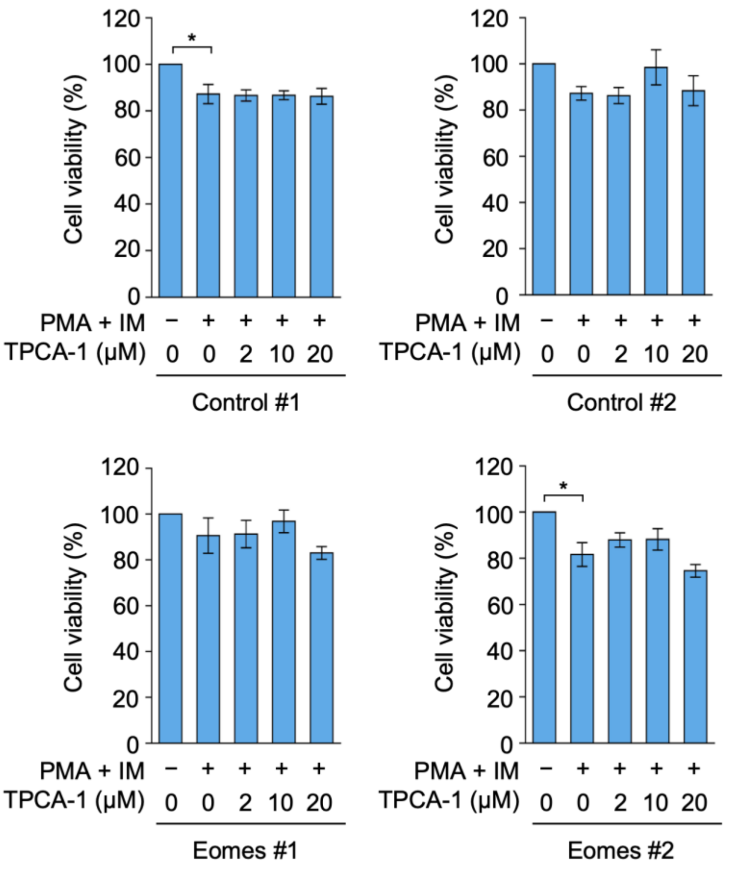

Figure S5. Effects of TPCA-1 on the cell viability of EL4 transfectants. Control #1, Control #2, Eomes #1, and Eomes #2 EL4 transfectants were pretreated with TPCA-1 for 1 h, and then treated with (+) or without (–) PMA (10 nM) and IM (1 μM) for 6 h in the presence or absence of TPCA-1 at the indicated final concentrations. Cell viability (%) is shown as the mean ± S.E. of three independent experiments. $^{*}P < 0.05$.

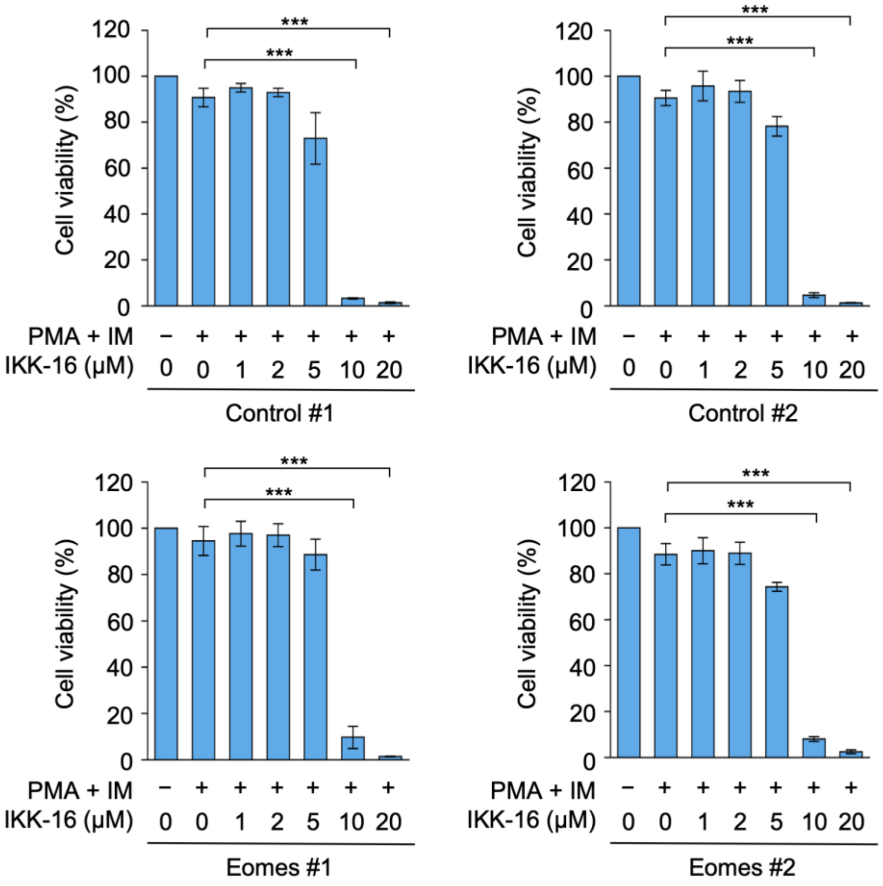

Figure S6. Effects of IKK-16 on the cell viability of EL4 transfectants. Control #1, Control #2, Eomes #1, and Eomes #2 EL4 transfectants were pretreated with IKK-16 for 1 h, and then treated with (+) or without (–) PMA (10 nM) and IM (1 μM) for 6 h in the presence or absence of IKK-16 at the indicated final concentrations. Cell viability (%) is shown as the mean ± S.E. of three independent experiments. $^{***}P < 0.001$.

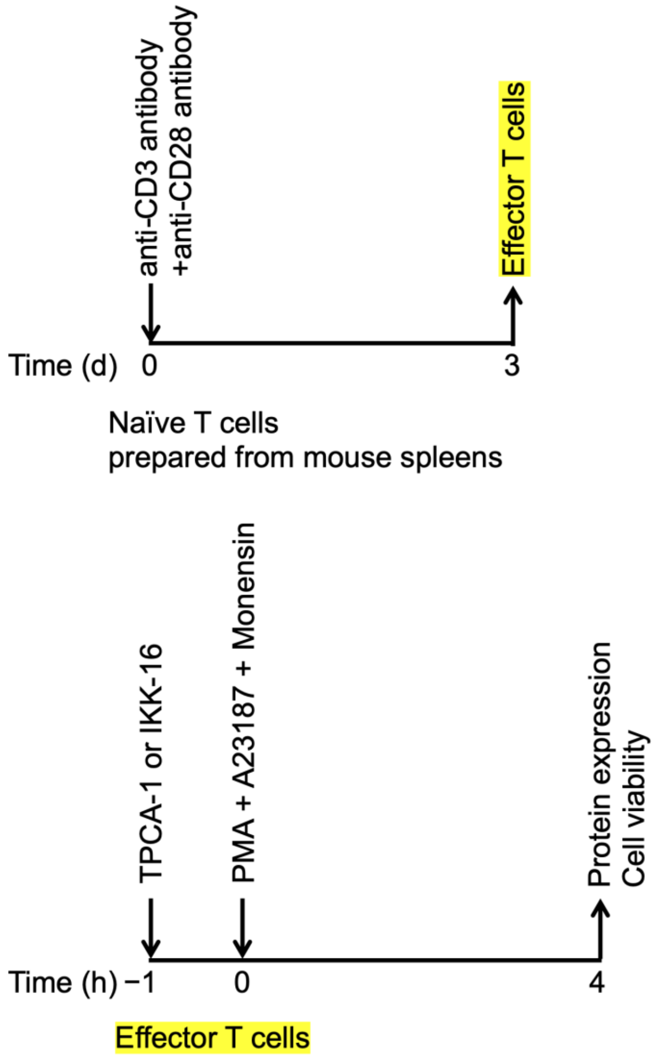

Figure S7. Experimental design for primary effector T cells. Naïve T cells were prepared from mouse spleens and incubated in plates coated with an anti-CD3 antibody (10 μg/mL) and anti-CD28 antibody (2 μg/mL) for 3 d. Effector T cells were harvested and preincubated with TPCA-1 or IKK-16 for 1 h, followed by an incubation with PMA (20 ng/mL) and A23187 (0.5 μg/mL) to mimic a TCR stimulation and monensin (2 μM) to block the secretion of cytokines for 4 h in the presence or absence of TPCA-1 or IKK-16. Cells were analyzed for the cell surface expression of CD4 and CD8α and intracellular expression of IFN-γ and IL-2 by flow cytometry.
